# History and Future Perspectives on the Discipline of Quantitative Systems Pharmacology Modeling and Its Applications

**DOI:** 10.3389/fphys.2021.637999

**Published:** 2021-03-25

**Authors:** Karim Azer, Chanchala D. Kaddi, Jeffrey S. Barrett, Jane P. F. Bai, Sean T. McQuade, Nathaniel J. Merrill, Benedetto Piccoli, Susana Neves-Zaph, Luca Marchetti, Rosario Lombardo, Silvia Parolo, Selva Rupa Christinal Immanuel, Nitin S. Baliga

**Affiliations:** ^1^Quantitative Sciences, Bill and Melinda Gates Medical Research Institute, Cambridge, MA, United States; ^2^Critical Path Institute, Tucson, AZ, United States; ^3^Office of Clinical Pharmacology, Center for Drug Evaluation and Research, U.S. Food and Drug Administration, Silver Spring, MD, United States; ^4^Center for Computational and Integrative Biology, Rutgers University, Camden, NJ, United States; ^5^Department of Mathematical Sciences and Center for Computational and Integrative Biology, Rutgers University, Camden, NJ, United States; ^6^Translational Disease Modeling, Data and Data Science, Sanofi, Bridgewater, NJ, United States; ^7^Fondazione the Microsoft Research – University of Trento Centre for Computational and Systems Biology (COSBI), Rovereto, Italy; ^8^Institute for Systems Biology, Seattle, WA, United States

**Keywords:** QSP modeling, systems biology, data science, systems pharmacology, bioinformatics, computational biology, drug development

## Abstract

Mathematical biology and pharmacology models have a long and rich history in the fields of medicine and physiology, impacting our understanding of disease mechanisms and the development of novel therapeutics. With an increased focus on the pharmacology application of system models and the advances in data science spanning mechanistic and empirical approaches, there is a significant opportunity and promise to leverage these advancements to enhance the development and application of the systems pharmacology field. In this paper, we will review milestones in the evolution of mathematical biology and pharmacology models, highlight some of the gaps and challenges in developing and applying systems pharmacology models, and provide a vision for an integrated strategy that leverages advances in adjacent fields to overcome these challenges.

## Introduction

Mathematical biology and pharmacology models are increasingly utilized in therapeutic and vaccine development, recognizing the need for improving the probability of success and/or reducing the cost of development. Quantitative systems pharmacology (QSP) models are being leveraged to aid in the identification of novel targets in early research, in the translational medicine activities for bringing molecules into the clinic, achieving proof of mechanism, and understanding of the variability in response to novel compounds in later clinical development. Bioinformatics and systems biology models and analyses present an opportunity to leverage big data for informing the development of the disease model scope in QSP, in addition to informing genotype-phenotype disease assessment, identifying responder/non-responder patient segments, or the development of novel biomarkers. With the appropriate investment in a mechanistic computational continuum, spanning bioinformatics, systems biology, and QSP, these computational approaches can pave the way for efficient and effective *in silico* drug discovery (e.g., identification of novel targets) or drug development (e.g., virtual human trials).

QSP is a mechanistic modeling approach that is used for the assessment of therapeutic intervention on a disease by linking descriptions of the molecular and cellular mechanisms of the disease and drug to system-wide dynamics, bridging biomarkers and clinical endpoints relevant for the disease (Gadkar et al., 2016; Balbas-Martinez et al., 2018; Kaddi et al., 2018; Coletti et al., 2020). Since they represent defined biological mechanisms, QSP models are suited to understanding the system-level response to treatment across multiple pharmacodynamic (PD) markers and clinical endpoints and to assessing the mechanistic basis for patient variability. There have been many QSP models published that address discovery and development questions across a variety of therapeutic areas, e.g., cardiovascular, cancer, immunology, oncology, and rare diseases, among others (Gadkar et al., 2016; Ming et al., 2017; Balbas-Martinez et al., 2018; Kaddi et al., 2018; Thiel et al., 2018; Abrams et al., 2020; Coletti et al., 2020). A recent industry-wide survey assessed current QSP support across therapeutic areas and provided insight into expected areas of future focus, including neuroscience and autoimmune disorders (Nijsen et al., 2018). Examples of the types of applications of QSP models across therapeutic areas include evaluation of the mechanism of action (MOA) of a new molecular entity, prediction of response in new populations or novel dosing paradigms, advancing hypotheses for a mechanistic basis of response/non-response, and exploration of MOA bases for synergies in combination therapy approaches (Ming et al., 2017; Bradshaw, 2019; Coletti et al., 2020).

Bioinformatics and systems biology offer a data-driven approach to the investigation of key mechanisms and associated networks implicated in disease pathophysiology that can subsequently inform the scope of biology represented in a QSP model. Moreover, the advancement of machine learning approaches is allowing more systematic knowledge discovery and extraction from the public domain, including published literature or public databases. The extracted knowledge base can be organized into logic-based or other computational frameworks that allow intermediate assessment or proof of concept for the modeling application at hand. These intermediate models can subsequently be translated into a QSP model framework for addressing the problems in question.

In this review, we will discuss the origin and evolution of QSP modeling and provide a vision for how QSP modeling can leverage advances in adjacent disciplines for an enhanced data‐ and knowledge-driven approach to model building and simulation.

## Historical Perspective and Example

### The QSP-Predecessor: Historical Overview of Mathematical Models of Physiology

**Figure 1:**
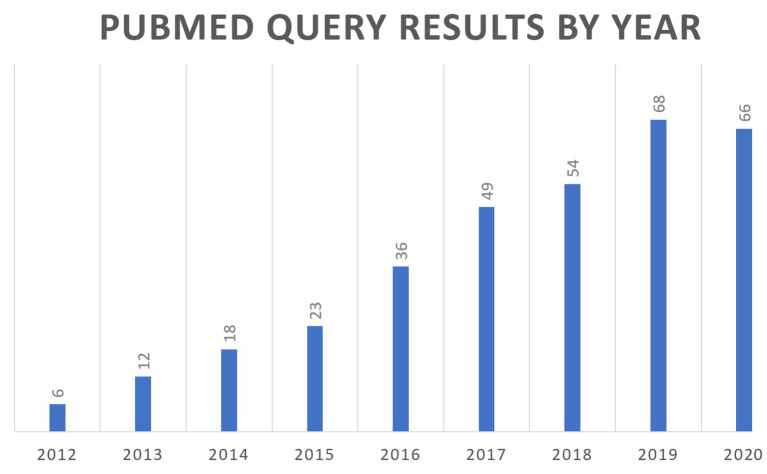
Review of PubMed citations by year for the search query (“systems pharmacology model”) OR (“quantitative systems pharmacology”) OR (“QSP model”).

In 2011, a National Institutes of Health (NIH) white paper authored by a group of scientists from the academia, industry, and government first coined the term quantitative and systems pharmacology (QSP), combining experimental and quantitative systems pharmacology approaches (Sorger et al., 2011). The goal was to define the ongoing merging of the fields of pharmacology and systems biology into a new discipline that could be applied across the drug discovery and development pipeline. The QSP proposed definition was “…an approach to translational medicine that combines computational and experimental methods to elucidate, validate and apply new pharmacological concepts to the development and use of small molecule and biologic drugs.” The QSP concept was a move away from the “one drug-one target-one pathway” paradigm to a more network-centric view of biology, with the central need to understand how a drug modulates molecular and cellular networks to impact pathophysiology. In addition, there was the parallel push for a quantitative framework to place the ever-increasing streams of biomedical data within the context of our current understanding of human physiology. QSP models are able to fulfill this need as they contain formal multiscale representations of human physiology and pathophysiology. These modeling efforts are quite valuable as they result in repositories of the present understanding of biological processes, and that can lead to identifying gaps and/or inconsistencies in our knowledge that require further experimental inquiry. Two recent reviews of QSP workflows are referenced here (Gadkar et al., 2016; Helmlinger et al., 2019). Figure 1 describes the growing number of references to QSP modeling in the scientific literature.

However, the origin story of QSP has deeper historical roots. QSP, and by proxy systems biology, can be traced back to its roots in classic models that pioneered the mathematical description of physiology. Mathematical models of disease have an established history in their utility for elucidating the biological and physiological mechanisms of disease. One of these seminal physiological models is Arthur Guyton’s (1919–2003) modeling of the circulatory system. Initially developed in the late 1960s, Guyton et al. represented in mathematical terms the human circulatory system (Guyton et al., 1972). This effort is considered an integral part for understanding the physiology of the cardiovascular system and diseases such as hypertension and heart failure. Guyton introduced concepts such as the key role of the kidney in long-term pressure regulation and the regulation of cardiac output by peripheral circulation, which were quite controversial at the time, but which have eventually become accepted dogma.

Despite being one of the earliest examples of integrated physiological modeling, Guyton’s modeling efforts display some of the features still present in today’s QSP models: nonlinear, multiscale, and modular. In fact, a look at the model scheme of his seminal work in 1972 shows a complex interconnected web of nodes reminiscent of a QSP scheme of today. The model or “systems analysis” as was referred to by Guyton, consisted of over 100 variables and hundreds of mathematical equations with the aim of representing the long-term regulation of blood pressure and cardiac output. It contained representations of 18 major systems that described the mechanical, neural, renal, and endocrine regulations that control the cardiovascular system. This was accomplished by subdividing the model into representations of organs and major vessels—accounting for fluids, electrolytes, and key hormonal regulation. Moreover, the model displayed emergence: the concept that high-level, integrative behavior of the system is not just the sum of the fundamental processes represented.

At the time, Guyton highlighted three points when describing the value of systems analysis that are still relevant when discussing QSP models:

1. Not every biological detail is needed or represented in fine detail in a model to accurately capture the gross behavior of the whole system.
2. Representing the system in a model allows the understanding of the importance of multiple regulatory processes acting on a single system.
3. Model building allows the evaluation of inconsistencies in data derived from different sources. In fact, Guyton’s efforts provided foundational experiments for understanding and elucidating circulatory physiology and control leveraged mathematical models to both understand and interpret experimental results. The iterative interplay between experiments and mathematical models exemplified by Guyton’s work is also a key feature of successful QSP models as new data informed mathematical models, which in turn were used for data interpretation and knowledge generation. The models are subsequently utilized in informing future experiments and their design in order to fill identified data and knowledge gaps.

### Advances in Measurement Technology and Data Science Provide Opportunity for Informing QSP Models

More than half a century later, systems-level data, revealing the complexity and multiscale nature of human biology and the pathophysiology of disease, are readily available due to the many incremental advances we have seen in measurement technology. The harnessing and contextualizing of these data for model development presents a significant opportunity in data sciences and modeling.

The advancement and maturation of measurement technologies, from the sequencing of the human and other organism’s genomes to technologies for measuring the other “-omes” (e.g., proteome, metabolome, epigenome, etc.), has had a dramatic impact on the capacity and potential of mechanistic biological models. Access to multiscale and mechanistic data enabled ambitious and forward-looking modeling initiatives such as the Physiome Project (Hunter and Borg, 2003). These efforts, in turn, have led to the establishment of repositories such as BioModels,^1^ the Physiome Model Repository,^2^ and the Drug Disease Modeling Resources Consortium,^3^ which are open to the community and host a variety of physiological models across scales (Le Novère et al., 2006; Sarwar et al., 2019). Depending on the scope of the original model and the problem context, these may be valuable resources for the development of QSP models (Weis et al., 2019). A further step which has been enabled by the advances in measurement systems are whole cell models. Examples under development for single-cell organisms may have relevance in infectious disease therapeutic development (Karr et al., 2012), and a consortium-based approach has been proposed for human whole-cell models (Szigeti et al., 2018). Lastly, the Human Cell Atlas Project (Regev et al., 2017) and similar disease-specific efforts integrate relevant knowledge at the cellular scale on human biology and disease pathophysiology, which lends key enabling information for mathematical biology and pharmacology (QSP) model development and application.

There are several steps that are necessary in the process of developing a QSP model. The first stage of knowledge discovery is the process of identifying and integrating multiscale biological knowledge on the disease of interest. At this stage, the modeler or team of modelers would typically interface with subject matter experts and harness published information from the literature on the disease. This activity is somewhat limited by the modeler’s time and ability to identify relevant articles of interest. A data science approach for knowledge discovery casts a much broader net over the entire space of published knowledge, both in published articles as well as public databases and model repositories. This allows a comprehensive review and integration of the available knowledge space and is the subject of the next section.

## An Industrial Renaissance, Opportunities For Advancing the Field: Turning Knowledge and Big Data Into Robust Models

### Importance of Knowledge Discovery: Developing Initial Model Structure and Building Knowledge and Data Libraries

The development of a QSP model requires an extensive effort to define the model structure required to satisfy the modeling goals. This task is usually based on a labor-intensive manual work carried out by a multidisciplinary team that defines the biological scope and identifies the components and interactions to include in the model. The complex nature of the biological systems makes the definition of the model scheme particularly challenging. The ultimate goal is to develop a parsimonious mathematical description of the biology for the determined modeling scope, and thus it is important to select only the most relevant biological entities and avoid the description of biological processes that are not informative.

The information to include in a model can be obtained from multiple sources. It can be derived from the literature, from biological databases, directly from the results of experiments carried out by the team that develops the model or, most commonly, by a combination of all these sources. It is noteworthy that experiments carried out in different conditions or with different protocols can yield conflicting findings. It is the task of the team involved in the model scheme definition to parse these data and establish appropriate computational protocols for incorporating them and to choose commensurate results. Moreover, the incomplete knowledge of the biological system often hampers its detailed mechanistic representation in mathematical terms, and it is thus necessary to make assumptions that are later tested using the model itself.

In this context, literature mining (French et al., 2015; Liu et al., 2015; Baker et al., 2017; Bachman et al., 2018) is one of the approaches supporting the definition of the model diagram and the identification of data sources and volumes, units of measurements, as well as parameter values and constraints. Once the desired scope of modeling has been clarified in terms of conditions, biological scales, and time resolution, the process goes through the collection of biological data and information. After expert interviews, the multidisciplinary team has a clear idea of the current understanding of biology and associated information that are yet to be identified. Critical information selection is important from this point on, and literature mining can be used for different purposes.

A first target of literature mining is the identification of review and research articles focused on understanding and describing the biological problem of interest. In this context, semantic-based searches are advantageous, especially when working in large multilevel or whole-body models. In contrast to standard keyword-based search methods, semantic searches allow for querying of concepts. Ontology-based approaches (Smith et al., 2007; Groß et al., 2016; Vitali et al., 2018), databases (Caspi et al., 2016; Olsen et al., 2017), and expert knowledge help craft extended domains of concepts made up of synonyms, alternatives, and related keywords each specifically addressing different areas of the modeled biology. Such concept-oriented approaches have been used, for example in (Michelini et al., 2018; Azer et al., 2019), allowing to screen for thousands of terms at once, dramatically widening the recall of the mining searches. At this stage, a first-pass screening allows the expert to identify the most promising literature and tune the queried concepts. The use of machine learning approaches for automatic annotation (Leaman et al., 2013, 2015; Wei et al., 2015) of the resulting queried literature offers new unbiased mentions of potentially relevant concepts appearing in the same articles.

Literature mining techniques can also be applied effectively to the modeling literature related to the biology of interest. Querying the literature for modeling concepts, along with any subset of the required biology, yields a corpus of modeling papers dealing with different subcomponents of the biological system. At this stage, an expanding set of text mining techniques greatly accelerates the extraction of mentions and appearances of kinetic and quantitative information from the modeling corpus.

The result of these two mining approaches is usually presented through a set of filters that identify how frequently specific concepts appear together and the original context where the concepts were found. The high levels of recall reached through the semantic-based search now pass through the funnel of natural language processing techniques to identify specific associations in the text. Common approaches include co-mention and linguistic relation analysis. Co-mention analysis can selectively extract the paragraphs where specific mentions of concepts appear. This approach may suffer from low precision which can be controlled *via* statistical enrichment of the co-mentioned concepts (Chen et al., 2017), increasing the entity recognition precision. While readily available, co-mention analysis is not able to capture a meaning in the presented results, making it ideal for summarization and knowledge classification purposes. For more specific knowledge extraction tasks, the linguistic relation analysis offers a more precise framework to analyze and mimic human language understanding of the text. This is done using rule-based methods and machine learning models to analyze the syntactic structure of the sentence and identify how relevant concepts relate to each other through meaningful linguistic structures in the sentence (Yu et al., 2018; Zhang et al., 2018).

**Figure 2:**
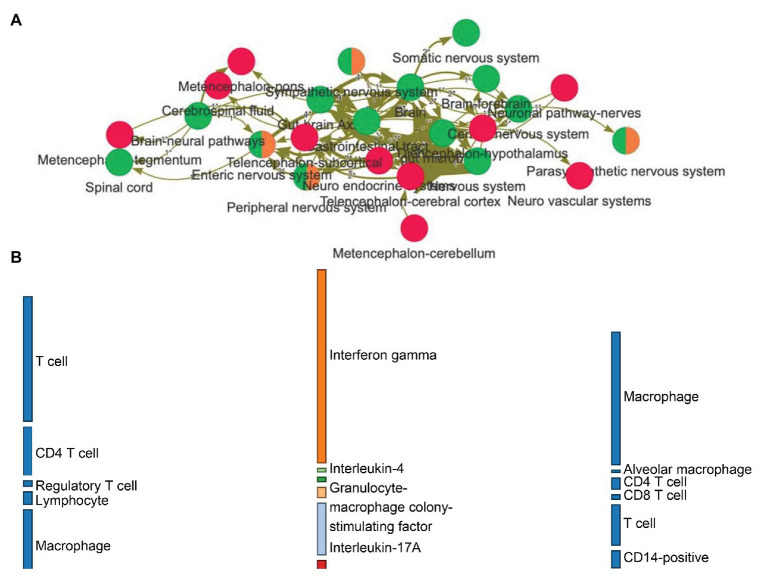
**(A)** Network visualization of how entities (nodes) relate to each other (edges) based on relations expressed in natural language. The frequency of a relation among entities can be represented with a varying thickness. *Red nodes* are central nervous system-related entities, *green ones* represent gut/brain-related annotations, while *orange nodes* have been classified into the peripheral nervous system. **(B)** The linguistic relations from the graph can be organized into a flow diagram, allowing the exploration of the directed effects between biological domains. This example makes visible how families of cells are interacting onto cytokines (*leftmost* to *middle* layers) and then how and what cytokines related back to the host cells (*middle* to *rightmost* layers).

A pair of concepts that may have been identified either *via* co-mention or relation analysis may appear multiple times in different contexts. By aggregating the occurrences of the same pair of concepts across all returned papers, it is possible to create a frequency map that links together concepts from multiple papers, offering a system view of the network connecting every identified concept in the literature, therefore facilitating the hypothesis generation process. Some of the ways of presenting and visualizing the concept network include:

- Interactive histogram, aiding in the assessment of frequency of single and combined occurrences of the terms in the selected literature.
- Network visualization. Nodes and edges represent entities and relations among those entities, respectively. The frequency of a relation among entities can be represented with a varying thickness (Figure 2A).
- Evidence sentence table. Beyond the relevant evidence text, additional information can be provided (e.g., impact factor, authors, year of publication, etc.). Linking the network visualization with the sentence table makes it possible to filter and navigate the concept network and have the associated evidence sentences tagged with entities.
- Bipartite information flow. This type of visualization allows exploring the (directed) relations between three domains of choice in a much more organized way than the network, showing the effect on varieties of cell families (Figure 2B).

The ability to leverage an evolving and advancing literature mining data science platform for biological modeling increases and improves the effectiveness of the modeling team and allows for a robust and data-driven knowledge discovery process of model development. Reutilizing previously built concept lists spanning diverse biological and modeling areas creates a virtuous feedback loop among the biological and data science team members. The developed text mining framework may at times become rich enough to evolve into stand-alone knowledge databases in specific areas (Azer et al., 2019; Essack et al., 2019), showcasing the effectiveness of linguistic structure analysis in enabling the discovery and identification of biological associations (Kveler et al., 2018).

As a further step toward a comprehensive knowledge of the biological system under investigation, the text mining-derived knowledge can be integrated with bioinformatics-derived findings. The number of publicly available omics datasets are constantly increasing, and they can be efficiently used to parameterize ordinary differential equation (ODE)-based mechanistic models (Fröhlich et al., 2018). Bioinformatics data-driven approaches using omics datasets for identifying the most active molecular interaction topologies, useful to draft the early components of the model diagram, are available as well (Nassiri et al., 2016).

### Incorporation of Literature Knowledge Into Logic-Based Models—Role of Automated Workflows, CS Tools (Languages, Computing), and Boolean Models

The information retrieval phase described above usually leads to a high volume of data that needs to be processed and organized to identify the components that are fundamental for modeling the biological system. The organization of the mined information in easily digestible ways with filters, graphical techniques, and accurately defined mining objectives is crucial to identifying the salient biological components to be modeled as well as the biological volumes, units of measurements, and parameters. Ideally, the former information represents minimal requirements to design and characterize a model. Moreover, it is important to evaluate whether there is enough data to parameterize the system. This review phase is fundamental because it unveils the knowledge gaps that need to be filled with new experiments and at the same time sets the basis for implementing the model.

Once the available data have been retrieved, the organization and efficient utility of this knowledge base comes into play. Logic models are an important tool in storing extracted knowledge in a therapeutic domain and in making this extracted knowledge “computable” and actionable. In addition to providing this computable knowledge notebook capability, logic models become a rapid mean of early feasibility assessment when the team is deciphering whether to embark on a QSP model development effort. When a decision is made to invest in building a QSP model, several approaches can be implemented, depending on the modeling scope and the available data. An intermediate step toward a fully quantitative description can be the implementation of or leveraging of an existing logic-based model that does not require an advanced mechanistic knowledge of the interactions among the components of the system. Indeed, logic-based models can be used to explore the qualitative properties of the behavior of a system and to address basic questions on the disease mechanism of therapeutic intervention. Agent-based models (ABMs), another example of a discrete modeling approach, employ a rule-based framework to describe the dynamics of heterogeneous systems of “agents,” which may represent individual cells, molecules, or other entities. ABMs and hybrid ABM approaches may be used in QSP applications to describe the emergence of disease states and spatially complex compartments, such as in solid tumors and granulomas (Cosgrove et al., 2015).

The simplest form of a logic model is a Boolean one in which the model scheme is translated into a network with variables that can have only two states, corresponding to the inactive and active conditions (Abou-Jaoudé et al., 2016). The variables are then updated by means of rules that can be interpreted as promotion or inhibition nodes connecting the regulating variables to the regulated ones. In step *i*, a regulative node is activated if its source variables are in the true state; otherwise, it is inactivated. Activated nodes are used to update the next state of system variables according to a wide variety of simulation strategies. Among them, the most popular simulation strategy assigns at step *i* + 1 a true value to all the variables that at step *i* do not have any active inhibition pointing to them and have at least one active promotion.

In some modeling applications, the two states offered by a Boolean description are not enough to provide an accurate qualitative representation of the system. In such cases, the Boolean model can be extended to a logic one, where variables can assume a wider range of integer values (for example three values, corresponding to “not expressed,” “low expression,” and “high expression”).

Once the logic model is constructed, it can be used to qualitatively explore the system dynamics, and in some applications, it can even constitute the final aim of the project (Traynard et al., 2017). Balbas-Martinez et al. for example, developed a Boolean system pharmacology model of inflammatory bowel disease that was used to test the effect of different immunotherapies (Balbas-Martinez et al., 2018). Conversely, when the final aim of the project is the development of a QSP model, the principal aim of logic models is to enable only a preliminary exploration of the model scheme and understand, *via* simulation, whether it provides a sufficiently accurate description of the system. In other words, it allows testing the reliability of the scheme and identifying possible knowledge gaps that could prevent the final implementation of the QSP model.

In addition to deterministic strategies, stochastic simulation strategies can be applied, which update the state of model variables in an asynchronous way by randomly selecting at each simulation step one regulative node to apply among those that are activated (Abou-Jaoudé et al., 2016). By combining the trajectories computed by multiple runs of stochastic simulations, the behavior of each variable can be explored in terms of the probability of being set in a specific state. This can be considered an intermediate step before a completely quantitative model simulation since it allows a preliminary comparison of the evolution of all model variables. The main limitation here is that the qualitative description offered by logic models does not allow linking the simulation step with a specific amount of time, and therefore the exploration of the model dynamics remains qualitative, mainly related to the analysis of the steady-state conditions, and cannot have the same level of detail offered by a quantitative model. Despite this, simulation results can be a valuable tool for discussing with experts and identifying inconsistencies between the model behavior and the available knowledge, especially when based on visual, easily interpretable, and mechanistic interaction dynamics. Any identified inconsistency will put the model scheme back under revision in order to understand with experts which parts to refine or extend. An additional phase of literature mining could also be executed to drive the model refinement.

Whether a Boolean, logical, or a full-fledged QSP model, the early stages of the model construction are iterative and incremental by nature, and computer science tools can facilitate the process. Dynamic architectures support the exploration and revision of the biological mining scope or the presentation of the mined results themselves. Bioinformatics tools are then useful in presenting interactive biological networks (Nassiri et al., 2016) that can be used as the building blocks of the model. There are visual modeling platforms (Funahashi et al., 2007; Lombardo and Priami, 2017) that can be especially useful in this phase when the model diagram is framed based on the literature mining results, datasets, expert knowledge, and hypotheses. A non-mediated graphical language makes the communication between different stakeholders easier and less error-prone. To populate the model with biological interactions among putative proteins, there are biological interactions manually curated from hundreds of different databases and collected in a single application programming interface (API)-based resource such as OmniPath (Türei et al., 2016).

As we have seen in this section, the knowledge discovery phase of model development can be significantly enabled through advanced data science application, specifically in the areas of knowledge extraction, data mining, and knowledge organization and assimilation. Moreover, the integration, organization, and distillation of this knowledge base make it amenable for incorporation into a QSP model framework and strategy. In conjunction with these applied approaches in the knowledge discovery phase of model development, a data-driven approach to leveraging bioinformatics data and systems biology networks to informing the multiscale components of QSP models and their associated model structure is an area of significant opportunity for the field. In the next section, we utilize tuberculosis (TB) to illustrate the application of bioinformatics and systems biology and discuss potential approaches for harnessing the outputs from these advanced technologies to advance a data-driven methodology for informing QSP models.

### Application of Bioinformatics and Systems Biology to Advancing QSP Modeling: An Example From Infectious Diseases Illustrating Where We Are and How Do We Bridge the Gap

TB, the disease caused by infection with *Mycobacterium tuberculosis* (MTB), is the leading cause of mortality from a single infectious agent worldwide, exceeding the mortality caused by HIV/AIDS. The 2019 Global Tuberculosis Report by the World Health Organization reported an estimated 10 million cases and nearly 1.5 million deaths in 2018 (WHO, 2019). While TB is treatable, the available therapies are burdensome. The standard regimen for a drug-sensitive disease, abbreviated HRZE, consists of four antibiotics taken daily for 6 months. The long duration of treatment makes non-adherence more likely, which in turn can promote the development of a drug-resistant disease. The development of shorter-duration, safe, and effective antibiotic regimens for TB is an urgent global need, and there are key opportunities for multiscale, mechanistic modeling efforts to contribute to this effort.

The granuloma, a hallmark of TB disease, typically contains a caseous core surrounded by T lymphocytes and macrophages (MFs) of different lineages (Flynn et al., 2011; Pai et al., 2016; Koch and Mizrahi, 2018). While the granuloma generates an immune microenvironment to control infection, it also provides MTB with a niche in which it can survive for prolonged periods (Ehlers and Schaible, 2012; Silva Miranda et al., 2012; Stanley and Cox, 2013). In a granuloma, MTB exists intracellularly within the host cells of different subtypes and extracellularly in the caseum, here onwards referred to as “compartments.” Because of the distinct microenvironments within these granuloma compartments, the bacteria exist as heterogeneous subpopulations with significant variability in growth characteristics and drug susceptibility (Lin et al., 2014; Marakalala et al., 2018; Cicchese et al., 2020). Therefore, characterizing the multiple layers of information processing networks (e.g., transcriptional, translational, metabolic, etc.) that underlie this heterogeneity and the variable drug susceptibility of the subpopulations may provide important compartment-specific information that can be used directly or aggregated to inform and advance multiscale QSP models that aim to simulate pharmacological response to combination therapy. The integration of molecular‐ and cellular-level modules of regulation and metabolism into the QSP framework may enable the rational formulation of effective combination drug regimens that have higher likelihoods of achieving clearance of heterogeneous populations of pathogen cells within the granuloma. We discuss below advances in the modeling and integration of metabolic and regulatory networks for MTB and the challenges in integrating these models with each other and within a QSP framework.

Constraint-based metabolic models (CBMs) enable genome-scale modeling of metabolic networks in the absence of kinetic parameters and provide a platform for integrating multi-omic datasets (Orth et al., 2010). While calculations from CBMs often result in a large solution space, which can include many biologically implausible solutions (Price et al., 2004; Schellenberger et al., 2011), the addition of biologically relevant constraints can significantly improve their predictive accuracy (Reed, 2012). Manually curated genome-scale CBMs have been constructed for many organisms (Oberhardt et al., 2009; Kim et al., 2012), including MTB. Specifically, three incrementally better models of MTB have been constructed incorporating progressively larger number of genes going from 661 genes mapped to 1,025 reactions (model iNJ661), to 810 genes mapped to 938 reactions (model iSM810), and to 1,011 genes mapped to 1,229 reactions (model iEK1011) (Jamshidi and Palsson, 2007; Ma et al., 2015; Kavvas et al., 2018). The performance of these models has been tested through the analysis of gene essentiality prediction using transposon sequencing (TNseq) as well as by assessing the accuracy of model-predicted growth characteristics (growth rate and biomass, etc.) in different culture media and conditions. Bordbar et al. introduced intracellular constraints on the metabolism of MTB in this model by limiting the nutrients accessible to the pathogen cells within the host environment by incorporating relevant nutrient exchange reactions (Bordbar et al., 2010). GranSim-CBM, which integrates a CBM with an ABM of granuloma formation, is an example of how such a multiscale approach can provide insight into MTB heterogeneity within the granuloma and provide guidance for experimental design, therapeutic selection, and performance targets (Pienaar et al., 2016).

However, metabolic models by themselves do not account for gene regulation, which is important to characterize granuloma compartment-specific phenotypes of MTB. To address this shortcoming, information regarding differential regulation of genes was integrated into the iNJ661 metabolic model (Chandrasekaran and Price, 2010) and subsequently into the iSM810 metabolic model (Ma et al., 2015) using the probabilistic regulation of metabolism (PROM) algorithm. While this integrated regulatory and metabolic network model made reasonable advancements in predicting the environment-specific growth phenotypes of MTB, it was based on a static protein-DNA interaction map for ~180 transcription factors (TFs), constructed by the overexpression of each TF in standard growth conditions (Minch et al., 2015). A more sophisticated approach for the inference and integration of a predictive gene regulatory network model with the metabolic model of MTB could significantly improve upon the performance of the PROM model to more accurately predict compartment-specific drug response phenotypes.

In order to understand how MTB responds and adapts to immunological attack, nutritional changes, and antitubercular treatment, we reconstructed a predictive model of an environment and gene regulatory influence network (EGRIN) model of MTB using a compendium of 2,325 publicly available transcriptome profiles (Peterson et al., 2014; Turkarslan et al., 2015). The microarray data were integrated with ~250,000 functional gene associations from STRING^4^ and nearly 5,000 operon prediction associations from MicrobesOnline^5^ using the *cMonkey* biclustering algorithm (Reiss et al., 2006) to identify sets of genes that are co-regulated over subsets of environmental conditions *via* shared TF-binding sequences, i.e., gene regulatory elements (GREs) in their promoters. Altogether, *cMonkey* incorporated 3,922 MTB genes (98% gene coverage) into 598 sets of putatively co-regulated genes (modules) and detected 1,192 GREs. This is the most comprehensive transcriptional regulatory network model for MTB that accurately predicts TF regulatory interactions and their downstream consequences (Peterson et al., 2016). This model represents a powerful means to uncover mechanisms by which MTB adapts to environmental conditions, including drug treatment. Further, the integrated deduced regulatory and metabolic (IDREAM) model was developed to integrate an EGRIN model with a CBM model, which has significantly improved performance in predicting environment-specific phenotypes (Wang et al., 2017). However, all of the previously developed methods [PROM (65, 68), rFBA; Covert et al., 2001, IDREAM; Wang et al., 2017, etc.] do not take into account one or more of four key properties of gene regulatory networks (GRNs) and motor neurons (MNs): (i) a single TF typically regulates dozens to several hundred genes in the genome; (ii) multiple metabolic genes can encode enzymes or enzyme subunits that catalyze the same reaction; (iii) a metabolic gene can be regulated by multiple TFs; and (iv) most importantly, the combinatorial regulatory scheme changes depending on the environmental context. Future work in integrating regulatory and metabolic networks will need to address these shortcomings to delineate how environment-specific combinatorial regulatory schemes constrain flux through metabolic reactions in each of the various compartments of the granuloma.

The integrated regulatory and metabolic network models can drive significant advancements of QSP models to predict drug effects on MTB phenotypes within granuloma compartments. By using a recently developed Path-seq technology (Peterson et al., 2019), it is now possible to enrich and quantify MTB transcriptomes in the caseum and MFs of different lineages [alveolar MFs (AMs), monocyte-derived MFs (MDMs), etc.] in the presence and absence of frontline drugs at model-predicted drug concentrations within each granuloma compartment. Similarly, using another recently developed technology, PerSort, drug-tolerant persister cell subpopulations can also be sorted for transcriptome, metabolome, and phenotype characterization (Srinivas et al., 2020). The nutrient composition and transcriptome profiles of host and pathogen populations (with and without drug treatment) within each granuloma compartment can then be used as inputs into the integrated regulatory and metabolic network model to predict growth rates as well as bactericidal and bacteriostatic drug effects on heterogeneous populations of pathogen cells. Finally, the differential drug effects on MTB cells across granuloma compartments can then be applied to predict the clearance rates of the pathogen.

In summary, bioinformatics and systems biology can make important contributions to QSP model development and application, enabling a data-driven framework for distilling relevant genotypic and phenotypic knowledge and data on disease pathophysiology and patient segments. This information is critical for assessing the impact of therapeutic interventions on patients’ phenotypes of interest using a mechanism-based simulation approach.

Parallel to the activities of informing a disease model structure and knowledge base is the development of a physiologically based pharmacokinetic (PBPK) model (Kuepfer et al., 2016). PBPK will describe the link between absorption, distribution, metabolism, and excretion (ADME) and the pharmacokinetic (PK) properties of a compound to the concentration of the molecular candidate in the represented physiological compartments of the QSP model. This allows the quantitative assessment and simulation of the local pharmacology of the therapy in each compartment represented in the QSP model. The development and history of PBPK models as stand-alone models and their utility in conjunction with disease models are the subject of the next section.

## Love-Hate Relationship with PBPK

QSP and PBPK models are intimately linked as PBPK models provide the pathway for the navigation of active drug entities within the physiologic system in which they elicit their effects. As such, PBPK models are an important tool for QSP models used to drive the local pharmacological response in the respective physiological compartments. Even though there is expected synergy of the models given their common mechanistic underpinnings, each is typically constructed adhering to the usual “fit-for-purpose” approach, with goals and objectives for use attached to the structure of the model and the data available and utilized for qualification. Table 1 compares the common purpose and/or goals of stand-alone PBPK and QSP models illustrating the common features and scope and those that are unique to the specific model types.

**Table 1:** Typical goals and objectives for physiologically based pharmacokinetic (PBPK) vs. quantitative systems pharmacology (QSP) models.

Model goals and objectives	Objective class	
	PBPK	QSP
FTIH dose prediction	1	2
DDI risk and specific drug interaction potential evaluation	1	N/A (PK interaction) 1 (PD interaction)
FTIP dose prediction	1	2
PK/PD evaluation	1	2
Formulation feasibility/performance evaluation	1	N/A
IVIVC	2	N/A
Biomarker-based evaluation	3	1
Weight/size impact on PK	1	3
Developmental influence on PK	1	3
Proof-of-mechanism evaluation	N/A	1
Proof-of-concept evaluation	N/A	1
Disease progression evaluation	N/A	1

1 = primary; 2 = complementary; 3 = secondary.

FTIH, first time in human; DDI, drug-drug interaction; FTIP, first in patient; PK, pharmacokinetics; PD, pharmacodynamics; IVIVC, *in vitro*-*in vivo* correlation; N/A, not applicable.

Most of the current overlap (real and perceived) in purpose is in the context of their use in drug development and the evolving landscape of commercial software solutions that have accelerated their utilization, expanded their use and scope, and grew both disciplines to the point where the skill set for model development of these types is in demand.

The origins of both model types (PBPK and QSP) are distinct, as are the historical disciplines that promoted their initial use. The first published PBPK models were primarily developed for pharmaceutical compounds in the 1970s, followed by those for environmental chemicals in the mid-1980s (Tan et al., 2018). Although the number of published models for environmental chemicals quickly outnumbered those for drugs, the latter has increased sharply more recently. Of the 1,313 references describing PBPK models from 1977 to 2016, the majority involved environmental chemicals (65%), followed by drugs (31%), with the remaining 4% involving endogenous compounds (e.g., monoclonal antibodies and small peptides; Tan et al., 2018). With respect to the authors of these works (PBPK models) the vast majority are coming from toxicology, environmental sciences, and pharmacy training. With respect to QSP models, the origins are more closely linked to pharmacology/bioengineering and a mechanistic understanding of disease progression and pathophysiology. Likewise, the early QSP application was more focused on hypothesis generation than to inform a regulatory decision. There is a tendency to set the QSP origin to the more recent NIH white paper (Sorger et al., 2011) in 2011, but this would be overlooking a rich history of system pharmacology models that existed prior to the declaration of the discipline, as has been described and outlined previously. Part of this disconnect with the dating also has to do with the training of the model developers, their choice for publication, and the rebranding of the discipline. The system pharmacology component of a QSP model requires the quantitative representation of all relevant reaction kinetics with enough complexity to achieve the purpose for which the QSP model is being designed to achieve. As the QSP representation is inherently multiscale from the pharmacology standpoint (organs → tissues → cells → proteins and regulatory metabolites → genes), the degree of mathematical and computational complexity is always understood as a key design element of the early-stage planning. On the PBPK side, the choice of complexity is also a consideration based on the expected physiologic space that the drug is expected to access.

Currently, both model types are actively employed to inform various stages of drug development and are somewhat linked by the commercial platforms and software utilized for their definition and analysis. At present, PBPK models are more clearly associated with regulatory deliverables having been the subject of regulatory guidance and advisory committees (FDA, 2019), although there is regulatory interest and support for continued investment in QSP models particularly to assist with proof-of-mechanism (POM) evaluation and proof-of-concept (POC) study design and attainment, endpoint definition, and pediatric extrapolation. With respect to the commercial software, this has been a concern as modelers trained in each discipline seek to extend their skills. A Venn diagram of the model space would suggest that QSP models envelop PBPK models, although PBPK modelers and software manufacturers tend to see QSP as an extension of PBPK modeling (Geerts et al., 2013). As this is an issue of approach only with concerns of how best to expand in each arena, there should be no concern. Software suitability and modeling approach relative to the necessary functionality, however, are another matter.

The development of commercial software for PBPK modeling has yielded professional solutions with many embedded features that also represent milestones in the evaluation of the approach, including normalization and scaling transformations that allow the accommodation of differences in *in vitro* systems for which key model inputs are reliant (Edginton et al., 2006; Rostami-Hodjegan and Tucker, 2007), unit conversion, flexibility in transport models dependent on a drug substance’s physiochemical properties, and physiologic parameter databases underlying the model’s structural parameters representing different species and human populations (Jamei et al., 2009; Pepin et al., 2021). All of this functionality is under the hood of a graphic user interface (GUI) that allows modelers and scientists to more efficiently specify requisite model features without the necessity of excessive coding and model checking. Of course, this comes at the expense of managing the various software updates and revisions, as there is a dedicated team of scientists/programmers that support the commercial development of these solutions. This solution works well for tasks which do not require extensive reworking of the model structure, of course. To be clear, the path forward when such conditions do arise is typically a request to the developer to expand the model structure or environment framework given the closed nature of the available solutions, necessitating time and expense to the modeler.

On the QSP front, this solution is not viable given the dynamic and unique nature of the models, their structure, and purpose. Likewise, there is less reliance on user interface-based model environment solutions and more utility of common programming-based solutions such as please change to R,^6^ MATLAB®, and Symbiology™ for QSP modeling. Of course, governance of coding contributions and the need for debugging and compiling are concerns, but these are deemed necessary overhead for the approach.

Hence, PBPK and QSP models are indeed intimately linked and are combined to inform *in silico* pharmacological interrogations. However, they also offer unique solutions to challenging problems in academic research and drug development and therefore should be recognized as distinct in their own right. Those who would embrace each model type must select the appropriate solution and environment to suit their purpose, as any modeler does.

## Model Calibration and Analysis: Evolving Technology and Methodological Advancement

Calibration of QSP models to data is essential to ensure that the model simulation outputs represent the behavior of the patient segment of interest and that the variability and uncertainty associated with the output reflect the commensurate level of confidence in the results. Access and availability of data, the size of the models, and the complexity brought about by the multiscale nature of the models make calibration a challenging task. Moreover, the disease status represented by the patient population reflects a steady-state condition or a point in time along the disease progression path. Clinical and preclinical data together represent key components of the disease and pharmacology that are necessary to capture into the QSP model.

At this point of the model development process, we have a model structure in place that has been informed by the knowledge discovery and informatics analyses that have been completed. The modeler needs to evaluate the appropriate size and scope of the QSP model given the range of data, mode of action of the therapeutic intervention, and the intended model application. Incorporating the mechanisms of disease that the therapeutic intervention is modulating aggregated to the level of the data available is necessary to ensure that calibration is even feasible. Identifying what are the system‐ vs. patient-specific parameters is also critical to developing a calibration strategy as well as a subset of parameters that will be fitted. Fitting all or most parameters is a failing strategy, especially given the number of parameters and the interdependencies between them. Taking into account the parameters that govern the steady-state conditions which define the patient segments of interest also plays into the calibration strategy. This may require some advanced methodology, as highlighted below using the linear-in-flux-expression (LIFE) methodology and described in more detail in (McQuade et al., 2017). Once a subset of parameters are identified that can be fitted, a number of optimization approaches can be deployed to identify an appropriate fit. In this section, we discuss the importance and need for evolving technology and methodological advances that are necessary to improve on the capability of QSP model optimization to clinical or preclinical data, as well as simulation strategies (Marchetti et al., 2017; Simoni et al., 2019a).

Multiscale QSP models rely on multiple sources of information to inform the pathophysiology, cellular, organ, and functional components of the disease, and its modulation under a therapeutic intervention. In order for QSP models to describe pathophysiological processes with sufficient granularity to address development questions, access to reliable, comprehensive, and open data sources to inform model parameters becomes increasingly essential. The availability of such domain sources for parameter values typically varies across modules of the overall QSP framework, with only some supporting high degrees of mechanistic detail. Access to literature-informed model parameters varies both across therapeutic areas and by biological scale. At the molecular or pathway scale, database resources like BRENDA (Jeske et al., 2019; www.brenda-enzymes.org) and Sabio-RK (Wittig et al., 2018) provide a wealth of information on the kinetics of enzyme-catalyzed reactions. Preclinical pharmacology data utilizing relevant disease biomarkers are often key data that can be incorporated early into the model development and are also useful for translational modeling work. Concordance of the biomarkers used in animal models and in the clinic is an advantage for the modeling work, but is not always the case given the differences between animal models and human diseases.

Certain parameters may be difficult to quantify or not directly measurable due to the inaccessibility of relevant samples, lack of assays, or because the parameters represent the lumped effects of processes that are not sufficiently understood. The latter situation is more frequently encountered at higher biological scales (e.g., tissue and organ levels or functional readouts like cognitive scores) where lack of knowledge of the many interacting processes encourages the use of more empirical frameworks to inform the organ sub-model and functional readouts incorporated into the multiscale model. In such situations, parameterization depends on the development of more effective optimization frameworks—for example, evolutionary approaches (Molina et al., 2018). In some cases, the goals of the QSP modeling effort may allow for avoiding parameterization issues altogether by employing network‐ or rule-based approaches (Bloomingdale et al., 2018) or utilizing machine learning or empirical modules as part of the model components. The availability of individual-level clinical data from the population of interest is necessary to represent the clinical phenotype of interest in the model, both at the pathophysiological and organ or functional levels. Clinical biomarker data are also key here to link mechanisms in the QSP model to the appropriate response variable that can reflect improvements in a disease with therapy. Additional clinical data, for example from disease registries, reflecting the disease progression trajectories of patients are especially important in some applications, where the population of interest is not at steady state prior to treatment. Combining multiple sources of clinical data with other types of data, for example from the literature and from preclinical species, represents a common challenge in informing QSP models. Advanced optimization and sensitivity analysis tools can help alleviate some of these concerns (Simoni et al., 2019b, 2020), especially as they pertain to the reproducibility of model outputs and model behavior (Kirouac et al., 2019). Additionally, tailoring and designing preclinical and clinical studies with the added objective of informing QSP and other models is also an important investment step to build increasing confidence in the models and the reproducibility of the model outputs.

One potentially powerful strategy that addresses some of these challenges is the development of disease platform QSP models—in other words, shifting away from the paradigm of developing individual models, with the associated steps, for individual therapeutics. A key advantage of QSP models, once developed, is that they serve as a repository of knowledge and data for the particular disease, and these knowledge and data can then be reused or repurposed. For example, they may be applied as a test bed for novel therapeutic or vaccine candidates for the same indication or, in certain cases, may be expanded to be applied for related diseases or patient segments. QSP modeling to support drug development for certain lysosomal storage diseases (LSDs) provides an example of the latter use case (Kaddi et al., 2018; Abrams et al., 2020). Platform model development introduces its own challenges and opportunities. Firstly, it can be necessary to reconcile alternative descriptions of processes. This is more prone to occur for modules at higher biological scales, such as the tissue or organ level, since those sub-models may not be as informed by mechanistic data and thus include more empirical descriptions. Secondly, the process of integration may necessitate the development of new modules to describe intermediate processes that were not included in component models, e.g., connecting or bridging molecular-scale processes to cellular‐ and organ-level outcomes fate. Finally, the integrated model must be re-qualified against all the data used to qualify the component models. This can be a computationally intensive and time-consuming process. However, once developed, an integrated platform QSP model can provide utility and insights beyond those of component models.

A number of advanced tools are available to elucidate the dynamic behavior of the model under different conditions. One such valuable tool is perturbation analysis (Abou-Jaoudé et al., 2016), which allows testing what happens to the model when the model scheme is perturbed to reproduce specific scenarios of interest. These scenarios could be linked to the variable state, for example the overexpression or knockdown of a specific model variable, or they can be linked to promotion/inhibition arcs, for example by changing the probability of selecting a specific node during stochastic simulation. The final result of this kind of analysis is a list of *in silico* evidence that can be used to refine the model scheme for prioritizing the next project steps. For example, if the perturbation analysis is showing that a part of the model is insensitive to any of the tested perturbations, then a possible model refinement is needed to obtain a simplified model scheme that will allow the implementation of a more parsimonious mathematical model while keeping similar accuracy in reproducing the considered simulation scenarios. On the other hand, if the perturbation analysis is showing that some variables are highly affected by the tested perturbations, these should be taken into consideration for model optimization. Specific literature search can be targeted to these variables in order to be sure of having included in the model scheme all the available knowledge. Moreover, ranked lists of these variables can be very valuable tools for prioritizing new experiments or to drive the implementation of the quantitative model, starting from the part of the network that shows the most promising and interesting behavior.

**Figure 3:**
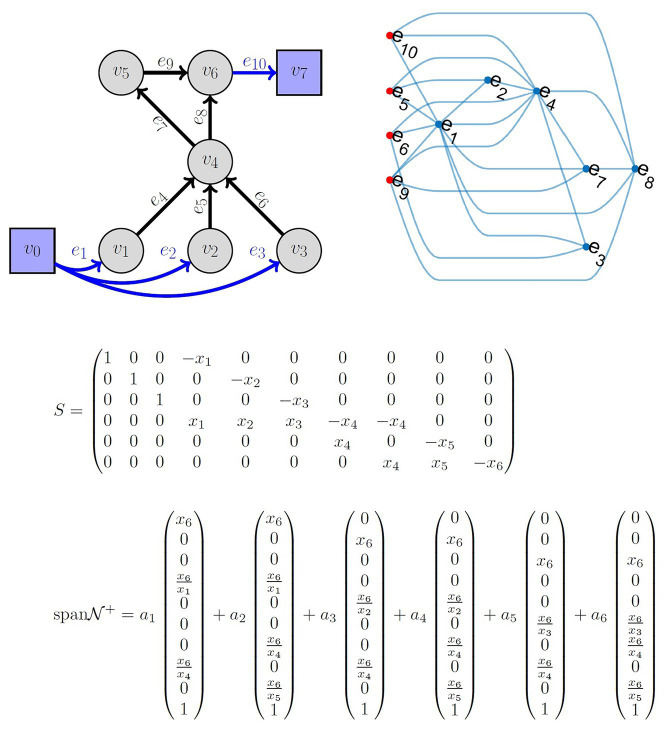
*Top left*: Example of a metabolic network known as reverse cholesterol transport in humans, described in reference McQuade et al. (2017). *Top right*: Graph of flux correlations. The nodes correspond to edges from the network. The relationships between fluxes in a cholesterol metabolism model at equilibrium. *Nodes in the graph* are fluxes. *Red nodes to the left* indicate independent fluxes and *blue nodes* are dependent fluxes. An *edge between a red node and another node* indicates that the expression for the dependent flux contains the independent flux. Dependent fluxes have edges between them when they share a dependent flux. *Middle*: The matrix *S* is the linear-in-flux-expression (LIFE) version of the stoichiometric matrix corresponding to the *top left network*. *x*_1_ corresponds to *v*_1_, etc. *Bottom*: The span of the positive null space is shown. Note that the nullity of *S* is 4. However, the positive basis requires six positively independent elements. The positive basis permits choosing *a_i_* ≥0 results in flux vectors that lie in the null space of *S* and the positive orthant.

Another important component of fitting QSP models is the tools that elucidate the underlying relationship among the model parameters and quantifying the algebraic relationships that govern information flow in the model. QSP models are often used with the hidden assumption that all fluxes are independent or have insignificant correlations (Schmidt et al., 2013; Allen et al., 2016; Hosseini and Gabhann, 2016), not leveraging the underlying model structure developed in the QSP model. Thus, the abundant know-how developed by systems biology (Palsson, 2015) and other research areas (Feinberg and Horn, 1974; Maeda et al., 1978; Biggs and Biggs, 1993; Jacquez and Simon, 1993; Caughman and Veerman, 2006; Klinke and Finley, 2012; Mirzaev and Gunawardena, 2013) is not translated into a large-scale use of such models (Sorger et al., 2011; Friedrich, 2016) as an *in silico* substitute for clinical trials. The LIFE approach (McQuade et al., 2017) allows the modeler to solve for the underlying relationship among the parameters in the model and quantify the flow of information under certain linearity criteria. This provides a subset of parameters at the source of these parameter networks, which can be used in a subsequent optimization strategy. The LIFE method begins by defining a network of biochemical reactions (metabolism) as a graph, where the nodes are reactants/products of metabolic reactions; the edge labels represent the reaction rates. In a classical systems biology approach, such a metabolic system is often written as *dx*/*dt* = *S*(*f*)*x*, where *x* is the vector of metabolite concentrations, *S* is the stoichiometric matrix, and *f* is the vector of fluxes. In the LIFE approach, the system is written as *dx*/*dt* = *S*′(*x*)*f*. The modified stoichiometric matrix *S*′(*x*) is a *n* × *m* matrix if the system has *n* metabolites and *m* fluxes, as opposed to ***S*** being *m* × *n*. For fixed metabolite levels, the relationship among fluxes is given by the null space of *S*(*x*) and the biologically viable ones by its intersection with the positive orthant. Therefore, for a system assumed to reach an equilibrium state, the positive basis vectors of the null space give a complete description of the flux parameters that maintain equilibrium. This allows both a better characterization of system equilibria and the possibility of simulating large system perturbations. An example of the LIFE approach and associated parameter network is shown in Figure 3.

In the previous sections, we have discussed the development and evolution of QSP models, with a detailed representation of the different phases of model building. Data science approaches are key to enabling the knowledge discovery phase of the initial phases of model development. Systems biology and bioinformatics provide data and context for the mechanisms that would subsequently be incorporated into the QSP framework. Novel advances and tools for analyzing QSP models and enabling robust model fitting to the data are essential in providing confidence in the model outputs and for the use of the models to make decisions. In this next section, we discuss the evolving landscape of the use of QSP models for decision making, both internally to an organization as well as for regulatory applications.

## Milestone Applications and Regulatory Interest and Buy-In

QSP modeling to inform various aspects of drug development is currently experiencing somewhat of a renaissance owing in part to the potential to link the mechanistic understanding of disease progression to biomarkers of interest and real-world data sources (e.g., various omics and patient-level clinical signs and symptoms) that can signal predisposition to response and may be useful to understanding the adverse events and perhaps off-target effects. Moreover, incorporation of real-world data may be useful for bridging efficacy to effectiveness assessment and the comparability of available therapies targeting similar patient populations. Further expansion and application of the discipline may also be useful to guide system vaccinology in a manner that guides future dose selection and identifies/corroborates meaningful correlates of protection (Rhodes et al., 2019). On the therapeutics front, there has been significant efforts in building and applying QSP models in the oncology and immunology disease areas, as triggered by industry-wide investments in these areas and unmet medical needs (Nijsen et al., 2018), highlighting the utility of QSP models to quantify the performance of combination pharmacology as well as for bridging pharmacology across adjacent yet distinct patient segments of disease.

QSP is part of the FDA’s Model-Informed Drug Development (MIDD) Pilot Program.^7^ The MIDD program is acknowledged in the sixth iteration of the Prescription Drug User Fee Act (PDUFA VI). Regulatory submissions of quantitative systems pharmacology modeling for applications in drug development have gradually increased over the past 10 years (Zineh, 2019), ranging from investigational new drug (IND) applications to new drug applications (NDAs) and biologic license applications (BLAs) and to efficacy supplements. QSP applications in regulatory submissions are broad, including toxicity prediction, dose selection, dosing regimen optimization, trial design, and clinical waiver.

It is recognized that model assessment should depend on the context of use (Ramanujan et al., 2019). The risk associated with decisions made based off a model and its prediction also depends on the context of application (use). Developing a QSP model to guide the design of *in vitro* vs. animal studies renders different levels of risk, with the former involving a lower decision risk. As a drug candidate moves into its clinical phases, the decision risks increase from the perspective of financial investment and human safety. Modelers intuitively know that no model can or intends to perfectly describe any specific system of human biology regardless of the complexity and size of a model as well as the amount of *in vitro* and *in vivo* data. Moreover, some of the challenges discussed in this paper regarding model calibration and informing the models with diverse datasets have raised questions on model result reproducibility. Therefore, moving forward, the best approach would be for the scientific community to address the need for rigorous criteria (Bai et al., 2019) and work toward common scientific expectations and best practices with respect to the assessment of QSP models and the criteria for evaluating and accepting their prediction in the context of various levels of decision risks. Moreover, additional emphasis in QSP model publications on model robustness, the reproducibility criteria utilized, and reliability of the model outputs presented can provide increased confidence in the community in the application of QSP modeling in drug research and development and reinforce our collective portfolio of success stories.

At the drug discovery or target validation stage, it is desirable for modelers to mechanistically explore to the greatest extent all possible pathways and feedback loops to propose and test a wide range of hypotheses in order to adequately capture all relevant human biology in as much detail as possible. Such efforts are made to delineate the effectiveness and toxicity profiles of a medical entity for the goal of minimizing the risk of safety issues or no clinical efficacy that would cause a development program to ultimately be terminated. On the other hand, the goal of applying QSP models in the late stages of drug development would not be to explore human biology associated with a target but would instead be used as a tool to define the dose/responses in the context of precision medicine. Considering *in vitro* and species differences in response to a medical entity which is under development for treating a disease, a model should be calibrated to as much its relevant clinical data as are available when the medical entity moves along its development phases. High scientific and statistical rigors for best practices are expected when QSP modeling is applied for late-stage drug development and beyond.

QSP modeling has a broad spectrum of applications, from discovery into late development. These applications provide a natural means of evolving a therapeutic area-wide QSP strategy that impacts discovery through development decisions within an organization. This strategy hinges on leveraging extensive knowledge discovery coupled with advanced data science and bioinformatics approaches in the early discovery stage of drug development. These computable knowledge and data, when integrated into a QSP platform, can aid with target elucidation and validation. As molecules advance into the preclinical stage, honing a more fit-for-purpose QSP strategy that benefits from basic pharmacology studies and more specific data on the pathways modulated by the molecular entity under development is a natural transition from an all-encompassing disease platform. This fit-for-purpose model also provides a means of utilizing translational strategies as the compound moves into the clinic to provide decision value for project teams on the path to clinical proof of concept. This QSP model is informed by clinical data and needs to be fitted to this data, as well as data that have been gleaned from earlier discovery and development efforts, to appropriately capture the disease trajectory of the relevant patient population to be assessed in clinical proof of concept and in late development. Finally, as candidate molecules enter late development, a data-driven and informed QSP modeling strategy can shed light on the precision medicine aspects of patient segment response or non-response and provide mechanistic underpinnings to variability in patient sub-segments. As the field of QSP modeling advances and the ability to leverage adjacent disciplines such as data science, bioinformatics, and systems biology and control continues to evolve and mature, the acceptability of QSP models for internal and regulatory decision making will increase, and more case studies showcasing these capabilities will emerge.

## Author Contributions

KA, JSB, JPFB, NSB, LM, and BP contributed to conception and design. All authors contributed to writing the manuscript and approved the final version. CK, LM, and RL prepared the figures.

### Conflict of Interest

The authors declare that the research was conducted in the absence of any commercial or financial relationships that could be construed as a potential conflict of interest.

## Footnotes

^1^www.biomodels.net

^2^https://models.physiomeproject.org/

^3^www.ddmore.eu

^4^https://string-db.org/

^5^http://www.microbesonline.org/

^6^https://www.R-project.org/

^7^https://www.fda.gov/drugs/development-resources/model-informed-drug-development-pilot-program

## References

[ref1] Abou-Jaoudé W. Traynard P. Monteiro P. T. Saez-Rodriguez J. Helikar T. Thieffry D.. (2016). Logical modeling and dynamical analysis of cellular networks. Front. Genet. 7:94. 10.3389/fgene.2016.00094, PMID: 27303434 PMC4885885

[ref89] Abrams R. Kaddi C. D. Tao M. Leiser R. J. Simoni G. Reali F.. (2020). A quantitative systems pharmacology model of Gaucher disease type 1 provides mechanistic insight into the response to substrate reduction therapy with eliglustat. CPT Pharmacometrics Syst. Pharmacol. 9, 374–383. 10.1002/psp4.12506, PMID: 32558397 PMC7376290

[ref2] Allen R. Rieger T. Musante C. (2016). Efficient generation and selection of virtual populations in quantitative systems pharmacology models. CPT Pharmacometrics Syst. Pharmacol. 5, 140–146. 10.1002/psp4.12063, PMID: 27069777 PMC4809626

[ref3] Azer K. Michelini S. Giampiccolo S. Parolo S. Leonardelli L. Lombardo R.. (2019). TB knowledgebase: interactive application for extracting knowledge from the TB literature to inform TB drug and vaccine development. Int. J. Tuberc. Lung Dis. 22:S592.

[ref4] Bachman J. A. Gyori B. M. Sorger P. K. (2018). FamPlex: a resource for entity recognition and relationship resolution of human protein families and complexes in biomedical text mining. BMC Bioinformatics 19:248. 10.1186/s12859-018-2211-5, PMID: 29954318 PMC6022344

[ref5] Bai J. P. F. Earp J. C. Pillai V. C. (2019). Translational quantitative systems pharmacology in drug development: from current landscape to good practices. AAPS J. 21:72. 10.1208/s12248-019-0339-5, PMID: 31161268

[ref6] Baker S. Ali I. Silins I. Pyysalo S. Guo Y. Högberg J.. (2017). Cancer hallmarks analytics tool (CHAT): a text mining approach to organize and evaluate scientific literature on cancer. Bioinformatics 33, 3973–3981. 10.1093/bioinformatics/btx454, PMID: 29036271 PMC5860084

[ref7] Balbas-Martinez V. Ruiz-Cerdá L. Irurzun-Arana I. González-García I. Vermeulen A. Gómez-Mantilla J. D.. (2018). A systems pharmacology model for inflammatory bowel disease. PLoS One 13:e0192949. 10.1371/journal.pone.0192949, PMID: 29513758 PMC5841748

[ref8] Biggs N. Biggs N. L. (1993). Algebraic graph theory, Vol. 67. Cambridge University Press.

[ref9] Bloomingdale P. Nguyen V. A. Niu J. Mager D. E. (2018). Boolean network modeling in systems pharmacology. J. Pharmacokinet. Pharmacodyn. 45, 159–180. 10.1007/s10928-017-9567-4, PMID: 29307099 PMC6531050

[ref10] Bordbar A. Lewis N. E. Schellenberger J. Palsson B. Ø. Jamshidi N. (2010). Insight into human alveolar macrophage and *M. tuberculosis* interactions via metabolic reconstructions. Mol. Syst. Biol. 6:422. 10.1038/msb.2010.68, PMID: 20959820 PMC2990636

[ref11] Bradshaw E. L. E. A. (2019). Applications of quantitative systems pharmacology in model-informed drug discovery: perspective on impact and opportunities. CPT Pharmacometrics Syst. Pharmacol. 8, 777–791. 10.1002/psp4.12463, PMID: 31535440 PMC6875708

[ref12] Caspi R. Billington R. Ferrer L. Foerster H. Fulcher C. A. Keseler I. M.. (2016). The MetaCyc database of metabolic pathways and enzymes and the BioCyc collection of pathway/genome databases. Nucleic Acids Res. 44, D471–D480. 10.1093/nar/gkv1164, PMID: 26527732 PMC4702838

[ref13] Caughman J. S. Veerman J. J. P. (2006). Kernels of directed graph Laplacians. Electron. J. Comb. 13:R39. 10.37236/1065

[ref14] Chandrasekaran S. Price N. D. (2010). Probabilistic integrative modeling of genome-scale metabolic and regulatory networks in *Escherichia coli* and *Mycobacterium tuberculosis*. Proc. Natl. Acad. Sci. U. S. A. 107, 17845–17850. 10.1073/pnas.1005139107, PMID: 20876091 PMC2955152

[ref15] Chen L. Zhang C. Wang Y. Li Y. Han Q. Yang H.. (2017). Data mining and pathway analysis of glucose-6-phosphate dehydrogenase with natural language processing. Mol. Med. Rep. 16, 1900–1910. 10.3892/mmr.2017.6785, PMID: 28627690 PMC5562079

[ref16] Cicchese J. M. Dartois V. Kirschner D. E. Linderman J. J. (2020). Both pharmacokinetic variability and granuloma heterogeneity impact the ability of the first-line antibiotics to sterilize tuberculosis granulomas. Front. Pharmacol. 11:333. 10.3389/fphar.2020.00333, PMID: 32265707 PMC7105635

[ref17] Coletti R. Leonardelli L. Parolo S. Marchetti L. (2020). A QSP model of prostate cancer immunotherapy to identify effective combination therapies. Sci. Rep. 10:9063. 10.1038/s41598-020-65590-0, PMID: 32493951 PMC7270132

[ref18] Cosgrove J. Butler J. Alden K. Read M. Kumar V. Cucurull-Sanchez L.. (2015). Agent-based modeling in systems pharmacology. CPT Pharmacometrics Syst Pharmacology 4, 615–629. 10.1002/psp4.12018, PMID: 26783498 PMC4716580

[ref19] Covert M. W. Schilling C. H. Palsson B. (2001). Regulation of gene expression in flux balance models of metabolism. J. Theor. Biol. 213, 73–88. 10.1006/jtbi.2001.2405, PMID: 11708855

[ref20] Edginton A. N. Schmitt W. Voith B. Willmann S. (2006). A mechanistic approach for the scaling of clearance in children. Clin. Pharmacokinet. 45, 683–704. 10.2165/00003088-200645070-00004, PMID: 16802850

[ref21] Ehlers S. Schaible U. E. (2012). The granuloma in tuberculosis: dynamics of a host-pathogen collusion. Front. Immunol. 3:411. 10.3389/fimmu.2012.00411, PMID: 23308075 PMC3538277

[ref22] Essack M. Salhi A. Stanimirovic J. Tifratene F. Raies A. B. Hungler A.. (2019). Literature-based enrichment insights into redox control of vascular biology. Oxidative Med. Cell. Longev. 2019:1769437. 10.1155/2019/1769437, PMID: 31223421 PMC6542245

[ref23] FDA (2019). Impact story: supporting drug development through physiologically based pharmacokinetic modeling. Available at: https://www.fda.gov/drugs/regulatory-science-action/impact-story-supporting-drug-development-through-physiologically-based-pharmacokinetic-modeling (Accessed February 22, 2019).

[ref24] Feinberg M. Horn F. J. M. (1974). Dynamics of open chemical systems and the algebraic structure of the underlying reaction network. Chem. Eng. Sci. 29, 775–787. 10.1016/0009-2509(74)80195-8

[ref25] Flynn J. L. Chan J. Lin P. L. (2011). Macrophages and control of granulomatous inflammation in tuberculosis. Mucosal Immunol. 4, 271–278. 10.1038/mi.2011.14, PMID: 21430653 PMC3311958

[ref26] French L. Liu P. Marais O. Koreman T. Tseng L. Lai A.. (2015). Text mining for neuroanatomy using WhiteText with an updated corpus and a new web application. Front. Neuroinform. 9:13. 10.3389/fninf.2015.00013, PMID: 26052282 PMC4439553

[ref27] Friedrich C. M. (2016). A model qualification method for mechanistic physiological QSP models to support model-informed drug development. CPT Pharmacometrics Syst. Pharmacol. 5, 43–53. 10.1002/psp4.12056, PMID: 26933515 PMC4761232

[ref28] Fröhlich F. Kessler T. Weindl D. Shadrin A. Schmiester L. Hache H.. (2018). Efficient parameter estimation enables the prediction of drug response using a mechanistic Pan-cancer pathway model. Cell Syst. 7, 567–579.e6. 10.1016/j.cels.2018.10.013, PMID: 30503647

[ref29] Funahashi A. Morohashi M. Matsuoka Y. Jouraku A. Kitano H. (2007). “CellDesigner: a graphical biological network editor and workbench interfacing simulator” in Introduction to systems biology. ed. Choi S. (Humana Press), 422–434.

[ref30] Gadkar K. Kirouac D. Parrott N. Ramanujan S. (2016). Quantitative systems pharmacology: a promising approach for translational pharmacology. Drug Discov. Today Technol. 21-22, 57–65. 10.1016/j.ddtec.2016.11.001, PMID: 27978989

[ref31] Geerts H. Spiros A. Roberts P. Carr R. (2013). Quantitative systems pharmacology as an extension of PK/PD modeling in CNS research and development. J. Pharmacokinet. Pharmacodyn. 40, 257–265. 10.1007/s10928-013-9297-1, PMID: 23338980

[ref35] Groß A. Pruski C. Rahm E. (2016). Evolution of biomedical ontologies and mappings: overview of recent approaches. Comput. Struct. Biotechnol. J. 14, 333–340. 10.1016/j.csbj.2016.08.002, PMID: 27642503 PMC5018063

[ref36] Guyton A. C. Coleman T. G. Granger H. J. (1972). Circulation: overall regulation. Annu. Rev. Physiol. 34, 13–44. 10.1146/annurev.ph.34.030172.000305, PMID: 4334846

[ref37] Helmlinger G. Sokolov V. Peskov K. Hallow K. M. Kosinsky Y. Voronova V.. (2019). Quantitative systems pharmacology: an exemplar model-building workflow with applications in cardiovascular, metabolic, and oncology drug development. CPT Pharmacometrics Syst. Pharmacol. 8, 380–395. 10.1002/psp4.12426, PMID: 31087533 PMC6617832

[ref38] Hosseini I. Gabhann F. M. (2016). Mechanistic models predict efficacy of CCR5-deficient stem cell transplants in HIV patient populations. CPT Pharmacometrics Syst. Pharmacol. 5, 82–90. 10.1002/psp4.12059, PMID: 26933519 PMC4761230

[ref39] Hunter P. J. Borg T. K. (2003). Integration from proteins to organs: the Physiome project. Nat. Rev. Mol. Cell Biol. 4, 237–243. 10.1038/nrm1054, PMID: 12612642

[ref40] Jacquez J. A. Simon C. P. (1993). Qualitative theory of compartmental systems. SIAM Rev. 35, 43–79. 10.1137/1035003 12387931

[ref41] Jamei M. Marciniak S. Feng K. Barnett A. Tucker G. Rostami-Hodjegan A. (2009). The Simcyp population-based ADME simulator. Expert Opin. Drug Metab. Toxicol. 5, 211–223. 10.1517/17425250802691074, PMID: 19199378

[ref42] Jamshidi N. Palsson B. Ø. (2007). Investigating the metabolic capabilities of *Mycobacterium tuberculosis* H37Rv using the in silico strain iNJ 661 and proposing alternative drug targets. BMC Syst. Biol. 1:26. 10.1186/1752-0509-1-26, PMID: 17555602 PMC1925256

[ref43] Jeske L. Placzek S. Schomburg I. Chang A. Schomburg D. (2019). BRENDA in 2019: a European ELIXIR core data resource. Nucleic Acids Res. 47, D542–d549. 10.1093/nar/gky1048, PMID: 30395242 PMC6323942

[ref44] Kaddi C. D. Niesner B. Baek R. Jasper P. Pappas J. Tolsma J.. (2018). Quantitative systems pharmacology modeling of acid Sphingomyelinase deficiency and the enzyme replacement therapy Olipudase Alfa is an innovative tool for linking pathophysiology and pharmacology. CPT Pharmacometrics Syst. Pharmacol. 7, 442–452. 10.1002/psp4.12304, PMID: 29920993 PMC6063739

[ref45] Kaddi C. Reali F. Marchetti L. Niesner B. Parolo S. Simoni G.. (2018). Integrated quantitative systems pharmacology (QSP) model of lysosomal diseases provides an innovative computational platform to support research and therapeutic development for the sphingolipidoses. Mol. Genet. Metab. 123, S73–S74. 10.1016/j.ymgme.2017.12.183

[ref46] Karr J. R. Sanghvi J. C. Macklin D. N. Gutschow M. V. Jacobs J. M. Bolival Jr B.. (2012). A whole-cell computational model predicts phenotype from genotype. Cell 150, 389–401. 10.1016/j.cell.2012.05.044, PMID: 22817898 PMC3413483

[ref47] Kavvas E. S. Seif Y. Yurkovich J. T. Norsigian C. Poudel S. Greenwald W. W.. (2018). Updated and standardized genome-scale reconstruction of *Mycobacterium tuberculosis* H37Rv, iEK1011, simulates flux states indicative of physiological conditions. BMC Syst. Biol. 12:25. 10.1186/s12918-018-0557-y, PMID: 29499714 PMC5834885

[ref48] Kim T. Y. Sohn S. B. Kim Y. B. Kim W. J. Lee S. Y. (2012). Recent advances in reconstruction and applications of genome-scale metabolic models. Curr. Opin. Biotechnol. 23, 617–623. 10.1016/j.copbio.2011.10.007, PMID: 22054827

[ref49] Kirouac D. C. Cicali B. Schmidt S. (2019). Reproducibility of quantitative systems pharmacology models: current challenges and future opportunities. CPT Pharmacometrics Syst. Pharmacol. 8, 205–210. 10.1002/psp4.12390, PMID: 30697975 PMC6482280

[ref50] Klinke D. J. 2nd Finley S. D. (2012). Timescale analysis of rule-based biochemical reaction networks. Biotechnol. Prog. 28, 33–44. 10.1002/btpr.704, PMID: 21954150 PMC3381993

[ref51] Koch A. Mizrahi V. (2018). Mycobacterium tuberculosis. Trends Microbiol. 26, 555–556. 10.1016/j.tim.2018.02.012, PMID: 29580884

[ref52] Kuepfer L. Niederalt C. Wendl T. Schlender J.-F. Willmann S. Lippert J.. (2016). Applied concepts in PBPK modeling: how to build a PBPK/PD model. CPT Pharmacometrics Syst. Pharmacol. 5, 516–531. 10.1002/psp4.12134, PMID: 27653238 PMC5080648

[ref53] Kveler K. Starosvetsky E. Ziv-Kenet A. Kalugny Y. Gorelik Y. Shalev-Malul G.. (2018). Immune-centric network of cytokines and cells in disease context identified by computational mining of PubMed. Nat. Biotechnol. 36, 651–659. 10.1038/nbt.4152, PMID: 29912209 PMC6035104

[ref54] Le Novère N. Bornstein B. Broicher A. Courtot M. Donizelli M. Dharuri H.. (2006). BioModels database: a free, centralized database of curated, published, quantitative kinetic models of biochemical and cellular systems. Nucleic Acids Res. 34, D689–D691. 10.1093/nar/gkj092, PMID: 16381960 PMC1347454

[ref55] Leaman R. Islamaj Doğan R. Lu Z. (2013). DNorm: disease name normalization with pairwise learning to rank. Bioinformatics 29, 2909–2917. 10.1093/bioinformatics/btt474, PMID: 23969135 PMC3810844

[ref56] Leaman R. Wei C.-H. Lu Z. (2015). tmChem: a high performance approach for chemical named entity recognition and normalization. J. Cheminform. 7:S3. 10.1186/1758-2946-7-S1-S3, PMID: 25810774 PMC4331693

[ref57] Lin P. L. Ford C. B. Teresa Coleman M. Myers A. J. Gawande R. Ioerger T.. (2014). Sterilization of granulomas is common in active and latent tuberculosis despite within-host variability in bacterial killing. Nat. Med. 20, 75–79. 10.1038/nm.3412, PMID: 24336248 PMC3947310

[ref58] Liu Y. Liang Y. Wishart D. (2015). PolySearch2: a significantly improved text-mining system for discovering associations between human diseases, genes, drugs, metabolites, toxins and more. Nucleic Acids Res. 43, W535–W542. 10.1093/nar/gkv383, PMID: 25925572 PMC4489268

[ref59] Lombardo R. Priami C. (2017). Graphical modeling meets systems pharmacology. Gene Regul. Syst. Bio. 11:1177625017691937. 10.1177/1177625017691937, PMID: 28469411 PMC5398309

[ref61] Ma S. Minch K. J. Rustad T. R. Hobbs S. Zhou S.-L. Sherman D. R.. (2015). Integrated modeling of gene regulatory and metabolic networks in *Mycobacterium tuberculosis*. PLoS Comput. Biol. 11:e1004543. 10.1371/journal.pcbi.1004543, PMID: 26618656 PMC4664399

[ref62] Maeda H. Kodama S. Ohta Y. (1978). Asymptotic behavior of nonlinear compartmental systems: nonoscillation and stability. IEEE Trans. Circ. Syst. 25, 372–378. 10.1109/TCS.1978.1084490

[ref63] Marakalala M. J. Martinez F. O. Plüddemann A. Gordon S. (2018). Macrophage heterogeneity in the Immunopathogenesis of tuberculosis. Front. Microbiol. 9:1028. 10.3389/fmicb.2018.01028, PMID: 29875747 PMC5974223

[ref60] Marchetti L. Priami C. Thanh V. H. (2017). Simulation algorithms for computational systems biology. Springer. 10.1002/wsbm.1459 31260191

[ref64] McQuade S. T. Abrams R. E. Barrett J. S. Piccoli B. Azer K. (2017). Linear-in-flux-expressions methodology: toward a robust mathematical framework for quantitative systems pharmacology simulators. Gene Regul. Syst. Biol. 11:1177625017711414. 10.1177/1177625017711414, PMID: 29581702 PMC5862386

[ref65] Michelini S. Balakrishnan B. Parolo S. Matone A. Mullaney J. A. Young W.. (2018). A reverse metabolic approach to weaning: in silico identification of immune-beneficial infant gut bacteria, mining their metabolism for prebiotic feeds and sourcing these feeds in the natural product space. Microbiome 6:171. 10.1186/s40168-018-0545-x, PMID: 30241567 PMC6151060

[ref66] Minch K. J. Rustad T. R. Peterson E. J. R. Winkler J. Reiss D. J. Ma S.. (2015). The DNA-binding network of *Mycobacterium tuberculosis*. Nat. Commun. 6:5829. 10.1038/ncomms6829, PMID: 25581030 PMC4301838

[ref67] Ming J. E. Abrams R. E. Bartlett D. W. Tao M. Nguyen T. Surks H.. (2017). A quantitative systems pharmacology platform to investigate the impact of Alirocumab and cholesterol-lowering therapies on lipid profiles and plaque characteristics. Gene Regul. Syst. Biol. 11:1177625017710941. 10.1177/1177625017710941, PMID: 28804243 PMC5484552

[ref68] Mirzaev I. Gunawardena J. (2013). Laplacian dynamics on general graphs. Bull. Math. Biol. 75, 2118–2149. 10.1007/s11538-013-9884-8, PMID: 24018536

[ref69] Molina D. LaTorre A. Herrera F. (2018). An insight into bio-inspired and evolutionary algorithms for global optimization: review, analysis, and lessons learnt over a decade of competitions. Cogn. Comput. 10, 517–544. 10.1007/s12559-018-9554-0

[ref70] Nassiri I. Lombardo R. Lauria M. Morine M. J. Moyseos P. Varma V.. (2016). Systems view of adipogenesis via novel omics-driven and tissue-specific activity scoring of network functional modules. Sci. Rep. 6:28851. 10.1038/srep28851, PMID: 27385551 PMC4935943

[ref71] Nijsen M. J. Wu F. Bansal L. Bradshaw-Pierce E. Chan J. R. Liederer B. M.. (2018). Preclinical QSP modeling in the pharmaceutical industry: an IQ consortium survey examining the current landscape. CPT Pharmacometrics Syst. Pharmacol. 7, 135–146. 10.1002/psp4.12282, PMID: 29349875 PMC5869550

[ref72] Oberhardt M. A. Palsson B. Ø. Papin J. A. (2009). Applications of genome-scale metabolic reconstructions. Mol. Syst. Biol. 5:320. 10.1038/msb.2009.77, PMID: 19888215 PMC2795471

[ref73] Olsen L. R. Tongchusak S. Lin H. Reinherz E. L. Brusic V. Zhang G. L.. (2017). TANTIGEN: a comprehensive database of tumor T cell antigens. Cancer Immunol. Immunother. 66, 731–735. 10.1007/s00262-017-1978-y, PMID: 28280852 PMC11028736

[ref74] Orth J. D. Thiele I. Palsson B. Ø. (2010). What is flux balance analysis? Nat. Biotechnol. 28, 245–248. 10.1038/nbt.1614, PMID: 20212490 PMC3108565

[ref75] Pai M. Behr M. A. Dowdy D. Dheda K. Divangahi M. Boehme C. C.. (2016). Tuberculosis. Nat. Rev. Dis. Primers. 2:16076. 10.1038/nrdp.2016.76, PMID: 27784885

[ref76] Palsson B. (2015). Systems biology. Cambridge University Press.

[ref77] Pepin X. J. H. Dressman J. Parrott N. Delvadia P. Mitra A. Zhang X.. (2021). In vitro biopredictive methods: a workshop summary report. J. Pharm. Sci. 110, 567–583. 10.1016/j.xphs.2020.09.021 32956678

[ref78] Peterson E. J. Bailo R. Rothchild A. C. Arrieta-Ortiz M. L. Kaur A. Pan M.. (2019). Path-seq identifies an essential mycolate remodeling program for mycobacterial host adaptation. Mol. Syst. Biol. 15:e8584. 10.15252/msb.20188584, PMID: 30833303 PMC6398593

[ref79] Peterson E. J. R. Ma S. Sherman D. R. Baliga N. S. (2016). Network analysis identifies Rv0324 and Rv0880 as regulators of bedaquiline tolerance in *Mycobacterium tuberculosis*. Nat. Microbiol. 1:16078. 10.1038/nmicrobiol.2016.78, PMID: 27573104 PMC5010021

[ref80] Peterson E. J. Reiss D. J. Turkarslan S. Minch K. J. Rustad T. Plaisier C. L.. (2014). A high-resolution network model for global gene regulation in *Mycobacterium tuberculosis*. Nucleic Acids Res. 42, 11291–11303. 10.1093/nar/gku777, PMID: 25232098 PMC4191388

[ref81] Pienaar E. Matern W. M. Linderman J. J. Bader J. S. Kirschner D. E. (2016). Multiscale model of *Mycobacterium tuberculosis* infection maps metabolite and gene perturbations to granuloma sterilization predictions. Infect. Immun. 84:1650. 10.1128/IAI.01438-15, PMID: 26975995 PMC4862722

[ref82] Price N. D. Reed J. L. Palsson B. (2004). Genome-scale models of microbial cells: evaluating the consequences of constraints. Nat. Rev. Microbiol. 2, 886–897. 10.1038/nrmicro1023, PMID: 15494745

[ref83] Ramanujan S. Chan J. R. Friedrich C. M. Thalhauser C. J. (2019). A flexible approach for context-dependent assessment of quantitative systems pharmacology models. CPT Pharmacometrics Syst. Pharmacol. 8, 340–343. 10.1002/psp4.12409, PMID: 30983158 PMC6617835

[ref84] Reed J. L. (2012). Shrinking the metabolic solution space using experimental datasets. PLoS Comput. Biol. 8:e1002662. 10.1371/journal.pcbi.1002662, PMID: 22956899 PMC3431291

[ref85] Regev A. Teichmann S. A. Lander E. S. Amit I. Benoist C. Birney E.. (2017). The human cell atlas. eLife 6:e27041. 10.7554/eLife.27041, PMID: 29206104 PMC5762154

[ref86] Reiss D. J. Baliga N. S. Bonneau R. (2006). Integrated biclustering of heterogeneous genome-wide datasets for the inference of global regulatory networks. BMC Bioinformatics 7:280. 10.1186/1471-2105-7-280, PMID: 16749936 PMC1502140

[ref87] Rhodes S. J. Knight G. M. Kirschner D. E. White R. G. Evans T. G. (2019). Dose finding for new vaccines: the role for immunostimulation/immunodynamic modeling. J. Theor. Biol. 465, 51–55. 10.1016/j.jtbi.2019.01.017, PMID: 30639297 PMC6860008

[ref88] Rostami-Hodjegan A. Tucker G. T. (2007). Simulation and prediction of in vivo drug metabolism in human populations from in vitro data. Nat. Rev. Drug Discov. 6, 140–148. 10.1038/nrd2173, PMID: 17268485

[ref90] Sarwar D. M. Kalbasi R. Gennari J. H. Carlson B. E. Neal M. L. de Bono B.. (2019). Model annotation and discovery with the physiome model repository. BMC Bioinformatics 20:457. 10.1186/s12859-019-2987-y, PMID: 31492098 PMC6731580

[ref91] Schellenberger J. Que R. Fleming R. M. T. Thiele I. Orth J. D. Feist A. M.. (2011). Quantitative prediction of cellular metabolism with constraint-based models: the COBRA toolbox v2.0. Nat. Protoc. 6, 1290–1307. 10.1038/nprot.2011.308, PMID: 21886097 PMC3319681

[ref92] Schmidt B. J. Casey F. P. Paterson T. Chan J. R. (2013). Alternate virtual populations elucidate the type I interferon signature predictive of the response to rituximab in rheumatoid arthritis. BMC bioinformatics 14:221. 10.1186/1471-2105-14-221, PMID: 23841912 PMC3717130

[ref93] Silva Miranda M. Breiman A. Allain S. Deknuydt F. Altare F. (2012). The tuberculous granuloma: an unsuccessful host defence mechanism providing a safety shelter for the bacteria? Clin. Dev. Immunol. 2012:139127. 10.1155/2012/139127, PMID: 22811737 PMC3395138

[ref34] Simoni G. Kaddi C. D. Tao M. Reali F. Tomasoni D. Priami C.. (2020). A robust computational pipeline for model-based and data-driven phenotype clustering. Bioinformatics btaa948. 10.1093/bioinformatics/btaa948, PMID: 33225350

[ref32] Simoni G. Reali F. Priami C. Marchetti L. (2019a). Stochastic simulation algorithms for computational systems biology: exact, approximate and hybrid methods. WIREs Syst. Biol. Med. 11:e1459. 10.1002/wsbm.1459, PMID: 31260191

[ref33] Simoni G. Thanh H. V. Priami C. Marchetti L. (2019b). A comparison of deterministic and stochastic approaches for sensitivity analysis in computational systems biology. Brief. Bioinform. 21, 527–540. 10.1093/bib/bbz014 30753281

[ref94] Smith B. Ashburner M. Rosse C. Bard J. Bug W. Ceusters W.. (2007). The OBO foundry: coordinated evolution of ontologies to support biomedical data integration. Nat. Biotechnol. 25, 1251–1255. 10.1038/nbt1346, PMID: 17989687 PMC2814061

[ref95] Sorger P. K. Allerheiligen S. R. B. Abernethy D. R. Altman R. B. Brouwer K. L. R. Califano A.. (2011). Quantitative and systems pharmacology in the post-genomic era: new approaches to discovering drugs and understanding therapeutic mechanisms. An NIH White Paper by the QSP Workshop Group; October 2011.

[ref96] Srinivas V. Arrieta-Ortiz M. L. Kaur A. Peterson E. J. R. Baliga N. S. (2020). PerSort facilitates characterization and elimination of persister subpopulation in mycobacteria. mSystems 5. 10.1128/mSystems.01127-20, PMID: 33262242 PMC7716392

[ref97] Stanley S. A. Cox J. S. (2013). Host-pathogen interactions during *Mycobacterium tuberculosis* infections. Curr. Top. Microbiol. Immunol. 374, 211–241. 10.1007/82_2013_332, PMID: 23881288

[ref98] Szigeti B. Roth Y. D. Sekar J. A. P. Goldberg A. P. Pochiraju S. C. Karr J. R. (2018). A blueprint for human whole-cell modeling. Curr. Opin. Syst. Biol. 7, 8–15. 10.1016/j.coisb.2017.10.005, PMID: 29806041 PMC5966287

[ref99] Tan Y. M. Worley R. R. Leonard J. A. Fisher J. W. (2018). Challenges associated with applying physiologically based pharmacokinetic modeling for public health decision-making. Toxicol. Sci. 162, 341–348. 10.1093/toxsci/kfy010, PMID: 29385573 PMC6084449

[ref100] Thiel C. Smit I. Baier V. Cordes H. Fabry B. Blank L. M.. (2018). Using quantitative systems pharmacology to evaluate the drug efficacy of COX-2 and 5-LOX inhibitors in therapeutic situations. NPJ Syst. Biol. Appl. 4:28. 10.1038/s41540-018-0062-3, PMID: 30083389 PMC6072773

[ref101] Traynard P. Tobalina L. Eduati F. Calzone L. Saez-Rodriguez J. (2017). Logic modeling in quantitative systems. Pharmacology 6, 499–511. 10.1002/psp4.12225, PMID: 28681552 PMC5572374

[ref102] Türei D. Korcsmáros T. Saez-Rodriguez J. (2016). OmniPath: guidelines and gateway for literature-curated signaling pathway resources. Nat. Methods 13, 966–967. 10.1038/nmeth.4077, PMID: 27898060

[ref103] Turkarslan S. Peterson E. J. R. Rustad T. R. Minch K. J. Reiss D. J. Morrison R.. (2015). A comprehensive map of genome-wide gene regulation in *Mycobacterium tuberculosis*. Sci. Data 2:150010. 10.1038/sdata.2015.10, PMID: 25977815 PMC4413241

[ref104] Vitali F. Lombardo R. Rivero D. Mattivi F. Franceschi P. Bordoni A.. (2018). ONS: an ontology for a standardized description of interventions and observational studies in nutrition. Genes Nutr. 13:12. 10.1186/s12263-018-0601-y, PMID: 29736190 PMC5928560

[ref105] Wang Z. Danziger S. A. Heavner B. D. Ma S. Smith J. J. Li S.. (2017). Combining inferred regulatory and reconstructed metabolic networks enhances phenotype prediction in yeast. PLoS Comput. Biol. 13:e1005489. 10.1371/journal.pcbi.1005489, PMID: 28520713 PMC5453602

[ref106] Wei C.-H. Kao H.-Y. Lu Z. (2015). GNormPlus: an integrative approach for tagging genes, gene families, and protein domains. Biomed. Res. Int. 2015:918710. 10.1155/2015/918710, PMID: 26380306 PMC4561873

[ref107] Weis M. Baillie R. Friedrich C. (2019). Considerations for adapting pre-existing mechanistic quantitative systems pharmacology models for new research contexts. Front. Pharmacol. 10:416. 10.3389/fphar.2019.00416, PMID: 31057411 PMC6482345

[ref108] WHO (2019). Global Tuberculosis Report: Executive Summary.

[ref109] Wittig U. Rey M. Weidemann A. Kania R. Müller W. (2018). SABIO-RK: an updated resource for manually curated biochemical reaction kinetics. Nucleic Acids Res. 46, D656–d660. 10.1093/nar/gkx1065, PMID: 29092055 PMC5753344

[ref110] Yu K. Lung P.-Y. Zhao T. Zhao P. Tseng Y.-Y. Zhang J.. (2018). Automatic extraction of protein-protein interactions using grammatical relationship graph. BMC Med. Inform. Decis. Mak. 18:42. 10.1186/s12911-018-0628-4, PMID: 30066644 PMC6069288

[ref111] Zhang Y. Lin H. Yang Z. Wang J. Zhang S. Sun Y.. (2018). A hybrid model based on neural networks for biomedical relation extraction. J. Biomed. Inform. 81, 83–92. 10.1016/j.jbi.2018.03.011, PMID: 29601989

[ref112] Zineh I. (2019). Quantitative systems pharmacology: a regulatory perspective on translation. CPT Pharmacometrics Syst. Pharmacol. 8, 336–339. 10.1002/psp4.12403, PMID: 30924594 PMC6618141

